# Supplementary material for: Cis-regulatory evolution spotlights species differences in the adaptive potential of gene expression plasticity
Source: Nat Commun. 2021 Jun 7;12:3376. doi: 10.1038/s41467-021-23558-2 (PMC8184852; doi:10.1038/s41467-021-23558-2)
Supplement: Supplementary file 1 — Supplementary Information [file 41467_2021_23558_MOESM1_ESM.pdf]

Supplementary information

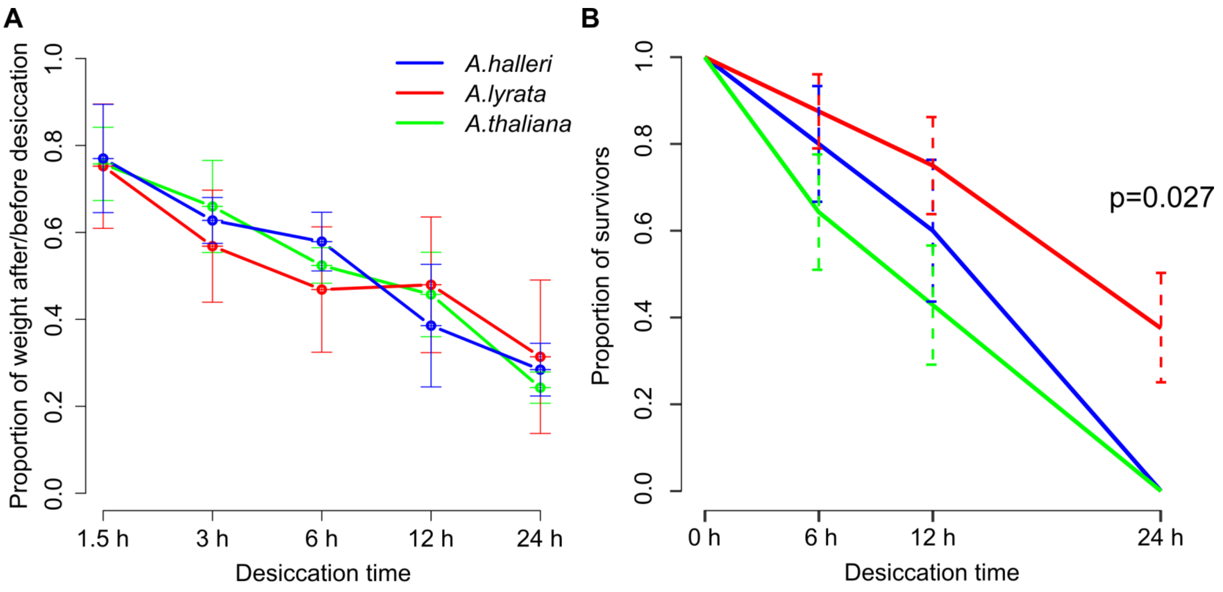

Supplementary Figure 1: (A) **The rate of water loss after 1.5 to 24h of desiccation is similar between the species.** The rate of water loss was inferred by the ratio of plant weight after / before the desiccation stress. Samples were weighted before the initiation of stress and immediately prior to RNA sampling. Species did not differ significantly in their rate of water loss (error bars indicate standard deviation, Interaction term species:duration of dehydration , F=0.562, *p*=0.803, glm model). Data shown in Supplementary Data 1.

(B) ***A. lyrata*** **is the most resistant to acute dehydration.** We performed the same dehydration experiment as for the transcriptome analysis, but instead of sampling material, we repotted the plants after 0h, 6h, 12h and 24h of dehydration and measured survival after 18 days. The survival rate after stress among species was significantly different and after 24h of dehydration, only *A. lyrata* survived (interaction term species:duration of dehydration in glm model, $F_{6,111}$= 8.71, *p*= 8.841e-08). This experiment included a representative set of 10, 17 and 14 genotypes of *A. halleri, A. lyrata* and *A. thaliana*, respectively (Supplementary Table 8). Data shown in Supplementary Data 2.

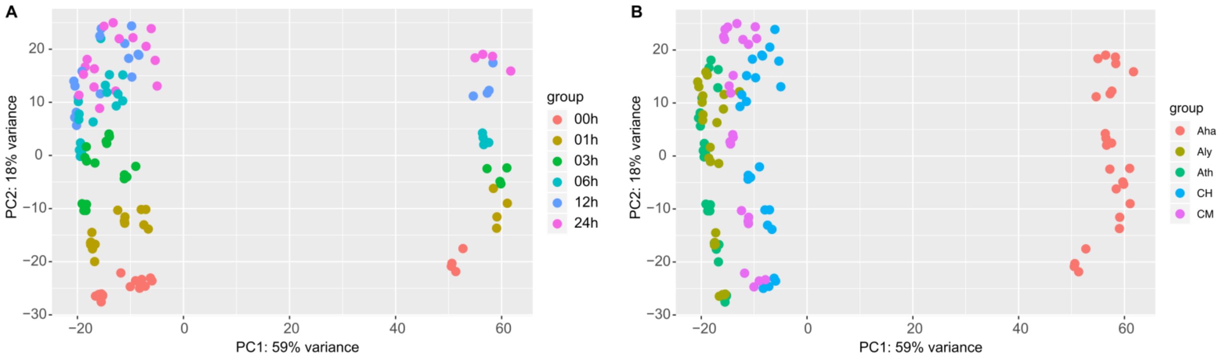

Supplementary Figure 2. **Differences between species and time points have a clear genetic basis.** Principal component analysis (PCA) of the 120 transcriptome samples collected over a desiccation time course for three species and two of their hybrids colored by A) time points and B) species (Aha – *A. halleri*, Aly – *A. lyrata*, Ath – *A. thaliana*, CH – F1 *A. thaliana* x *A. halleri*, CM – F1 *A. thaliana* x *A. lyrata*). The PCA separates the samples by species (PC1 59% variance) and by time-points (PC2 18% variance).

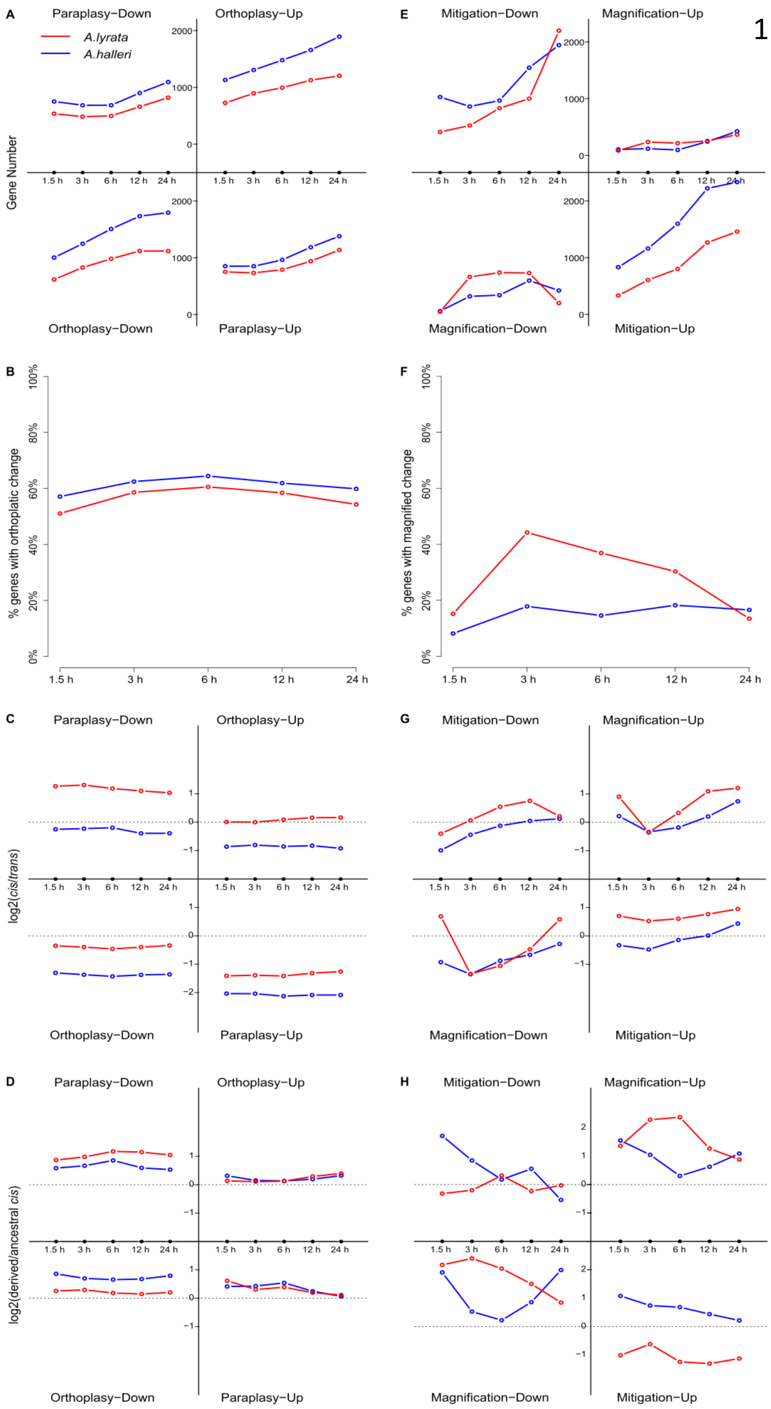

Supplementary Figure 3. **Extension of Figure 2 and 3 to all time points of the experiment.** Basal changes (A – D) and plastic responses to the stress (E – H) in *A. lyrata or A. halleri*, compared *to A. thaliana*. (A) The number of genes with a basal change in the direction (ortho-) or opposite (para-) to the direction of the stress reaction in *A. thaliana* increases over the time course. (B) The proportion of genes with an orthoplastic change is not dependent on the time point at which plasticity is determined. (C) The proportion of basal changes explained by *cis*-acting differences is not dependent on the time point at which plasticity is determined. (D) The proportion of basal changes explained by a derived *cis* is not dependent on the time point at which plasticity is observed. E) The number of genes with a mitigated response increase during the time course. The number of genes with a magnified response do not increase to the same extent. (F) In *A. halleri*, the proportion of genes with a magnified response is not dependent on the time point, whereas in *A. halleri* the proportion of magnified genes is increased between 6 and 12 hours. (G) The proportion of responses to the stress explained by plastic *cis*-acting differences is dependent on the time point in *A. lyrata*. H) The proportion of responses to the stress explained by derived *cis*-acting changes is dependent on the time point.

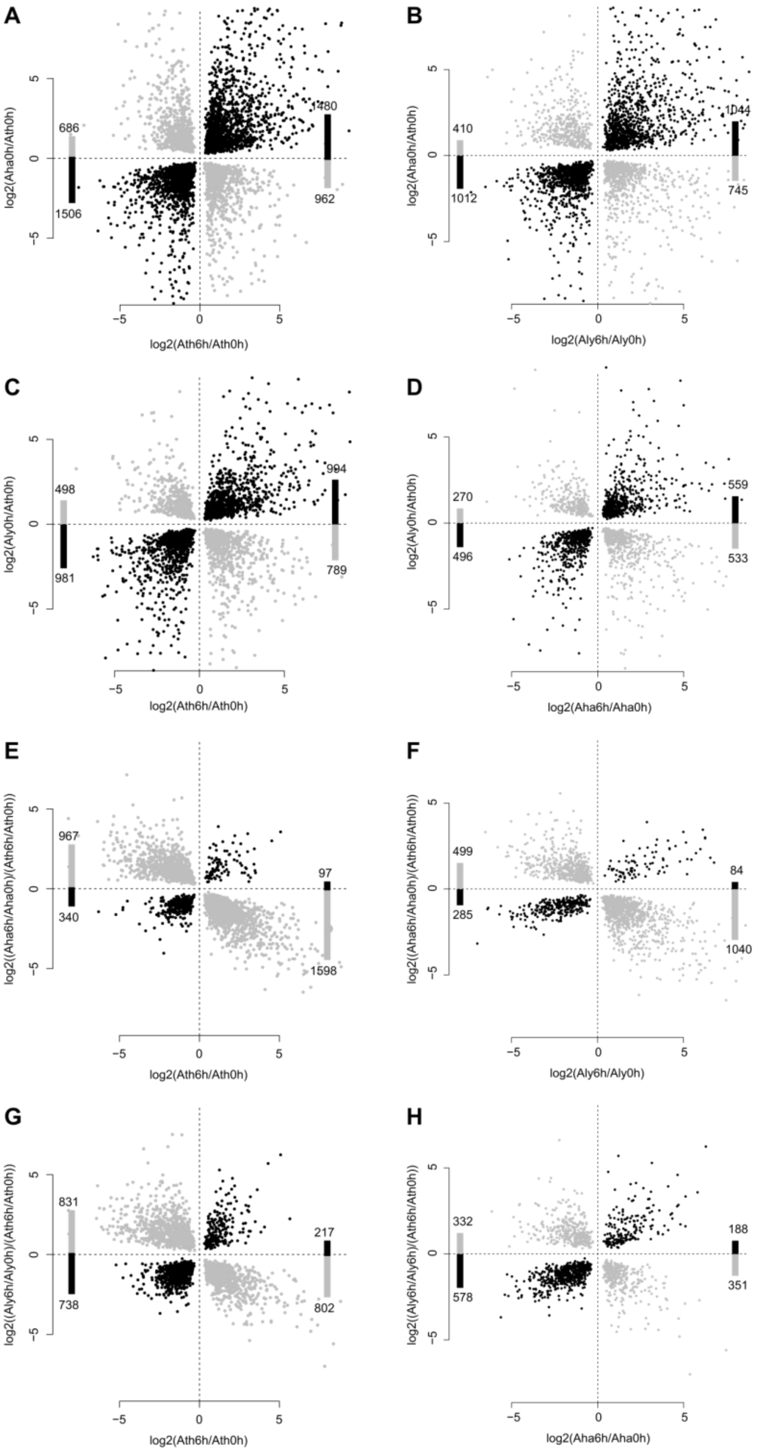

Supplementary Figure 4. Orthoplastic evolution of basal (A – D) gene expression in *A. lyrata* or *A. halleri* is robust to the species used for comparison. A mitigated response to stress in *A. halleri* is robust to the proxy used for plastic changes (E – F) . This is not the case in *A. lyrata* (G-H). A-B: Basal changes in *A. halleri* vs *A. thaliana* using *A. thaliana* (A) and *A. lyrata* (B) as a comparison.

C-D: Basal changes in *A. lyrata* vs *A. thaliana* using *A. thaliana* (C) and *A. halleri* (D) as a comparison.

E-F: Plastic changes in *A. halleri* using *A. thaliana* (E) and *A. lyrata* (F) as a comparison.

G-H: Plastic changes in *A. lyrata* using *A. thaliana* (E) and *A. halleri* (F) as a comparison.

A-D: orthoplastic: black, paraplastic: grey

E-H: magnification: black, mitigation: grey

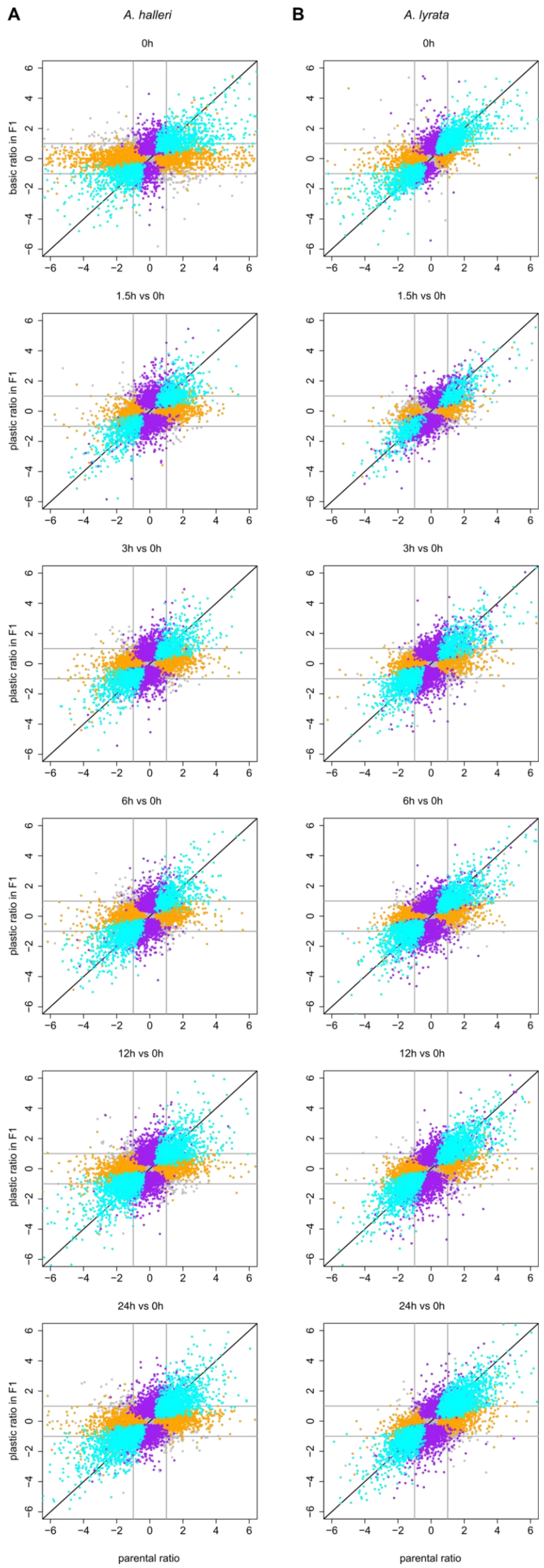

Supplementary Figure 5. ***Cis* regulatory changes explain a large part of the interspecific differences between the species.** (A) *A. halleri*. (B) *A. lyrata*. Cyan: *cis* change explains most of the parental difference; Orange parental difference are not associated with a bias in allele specific expression and thus assumed to be controlled in trans- (no significant *cis*-acting change); purple: Significant cis-acting changes are detected but the difference between parents is not significant. The allele-specific expression ratio of purple genes is however strongly correlated with the parental expression ratio, indicating that this class of genes reflect lower power for detecting parental differences (shown in Supplementary Table 8).

At 0h, the genetic basis of regulatory differences in basal expression is shown by plotting log2ratio of *A. halleri*/*A. thaliana* or *A. lyrata*/*A. thaliana* of alleles in the hybrid (y-axis) against the ratio of parental expression (x-axis). From 1.5h to 24h, the genetic basis of regulatory changes is determined by comparing ratio of log2 ratios at xh vs. 0h for *A. halleri*/*A. thaliana* or *A. lyrata*/*A. thaliana* alleles in hybrid (y-axis) with parents (x-axis) at each time point.

Pearson correlations for *cis* (cyan), non-*cis* (trans; orange) and purple (compensatory; *cis*-change but no significant parental difference) are shown in Supplementary Table 8.

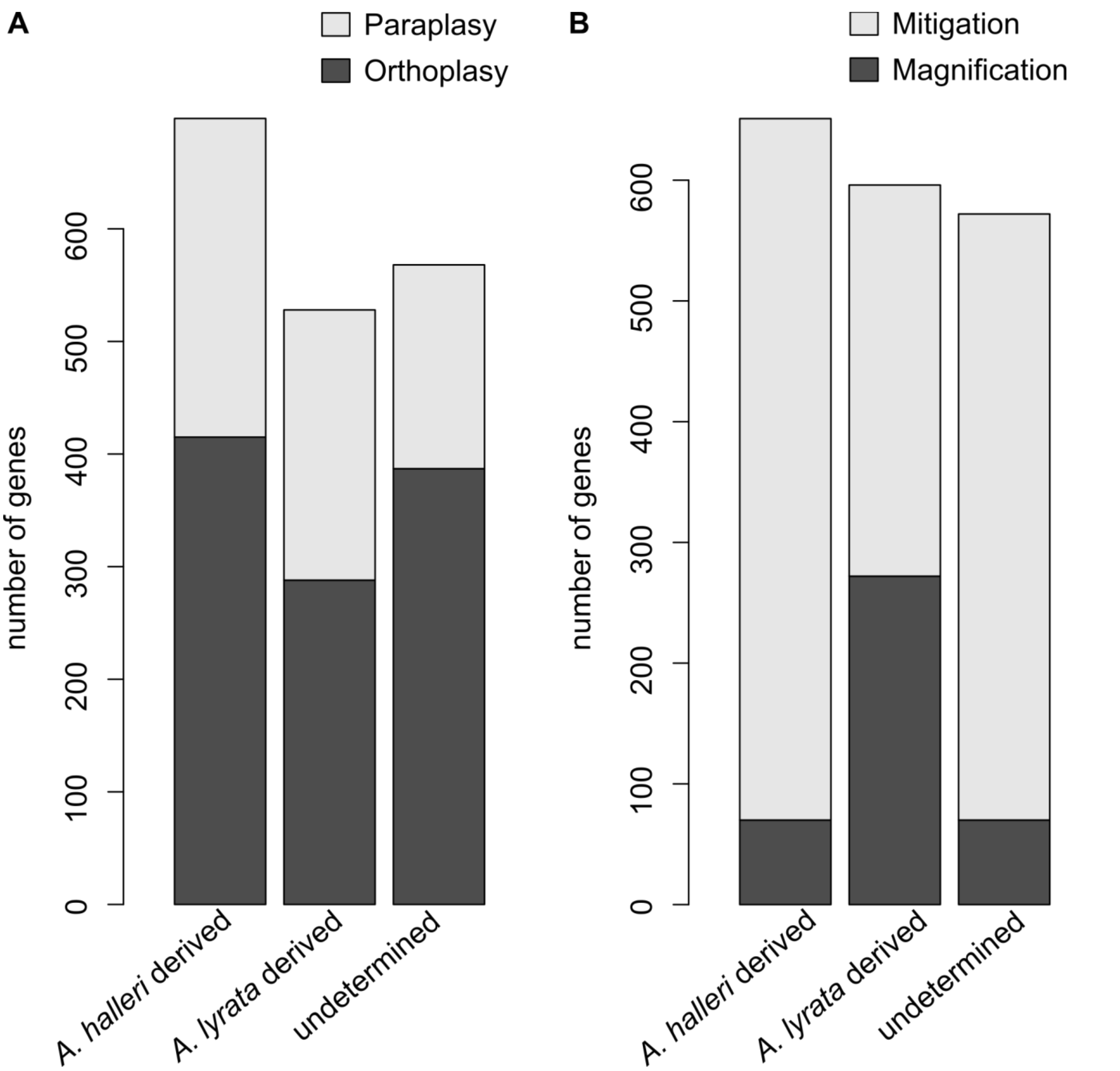

Supplementary Figure 6. **The total number of genes with derived and undetermined *cis*-regulatory changes are similar.** The number of genes that have (A) a basal change and either an undetermined or derived *cis*-regulatory change or a (B) plastic response and either an undetermined or derived *cis*-regulatory change. Genes are partitioned by the direction of the cis-acting effect detected in basal expression (A) or in the response slope (B). The proportions from Figure 4C are based on these numbers. Because the direction of effect of cis-regulatory changes of undetermined origin cannot be ascertained, the number of genes with these changes are not partitioned functionally.

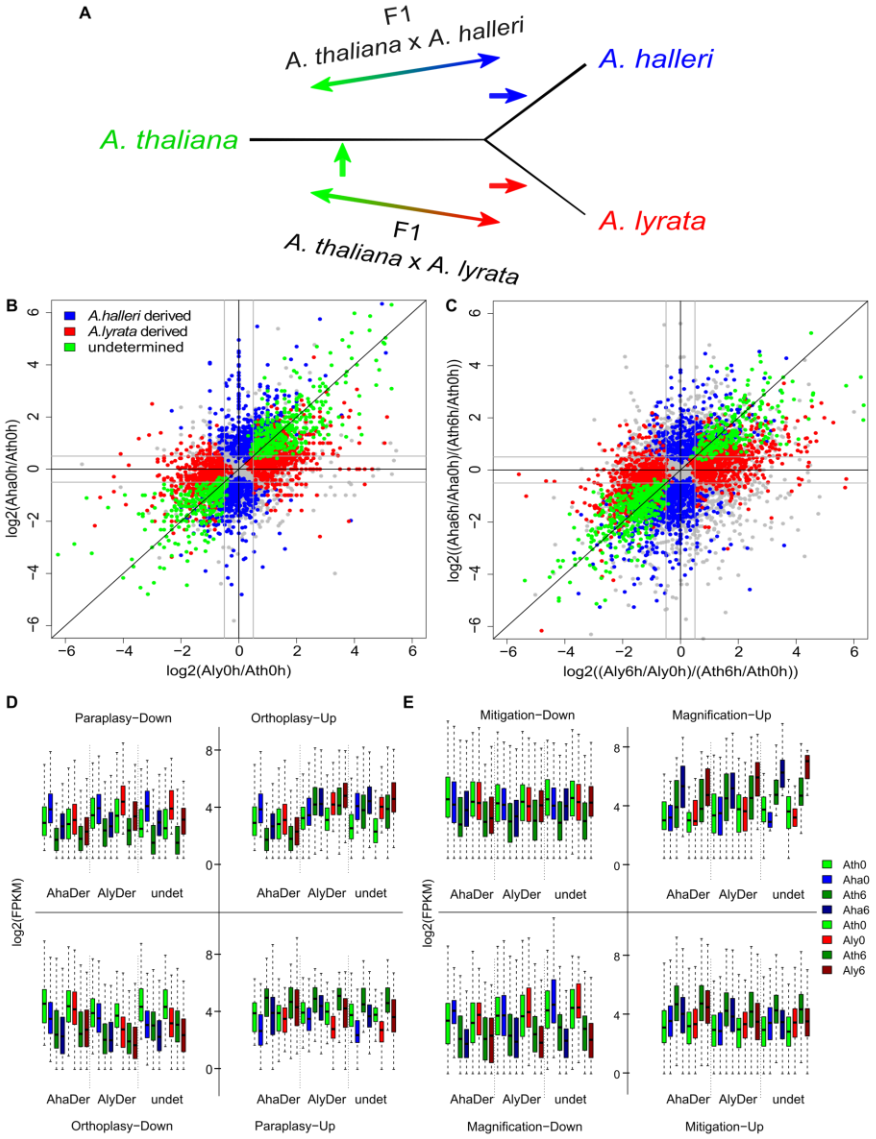

Supplementary Figure 7. **The phylogenetic origin of cis-acting changes was determined based on the comparison of allele-specific expression ratios in the two F1 hybrids *A. thaliana-A. lyrata* and *A. thaliana-A. halleri*.** (A) Phylogenetic relationship between the three species allowed distinguishing cis-acting changes of undetermined origin (green arrow), which are shared in the two F1 hybrids, from the derived *cis*-acting changes, which are specific (blue and red arrow). Also shown in Figure 4B. (B) basal and (C) plastic cis-acting changes. Log2 allelic ratios in AthxAha hybrids plotted against log2 allelic ratios in AthxAly hybrids in basal cis mutations (B), genes with an undetermined *cis*-regulatory change are on the diagonal (green), genes with a derived *cis*-regulatory change in *Arabidopsis lyrata* are on the horizontal central lines (red) and genes with a derived *cis*-regulatory change in *A. halleri* are on the vertical line (blue). Log2 of allelic ratio changes between 6h and 0h in AthxAha hybrids plotted against log2 allelic ratio changes in AthxAly hybrids in plastic cis mutations

(C). Box and whiskers depict the 75th and 95th interquantile ranges, respectively, dots show outliers.

The inferred phylogenetic inference of derived *cis*-acting changes is independent of read count levels. D). The total expression level before stress for the orthoplastic and paraplastic genes in the F1 hybrids. Dark colors are for time point 6h, light colors for time point 0 hours. *A. thaliana*: green, *A. halleri*: blue and *A. lyrata*: red. E). The total expression level before and after stress for the magnified and mitigated genes in the F1 hybrids. Dark colors are for time point 6h, light colors for time point 0 hours. *A. thaliana*: green, *A. halleri*: blue and *A. lyrata*: red.

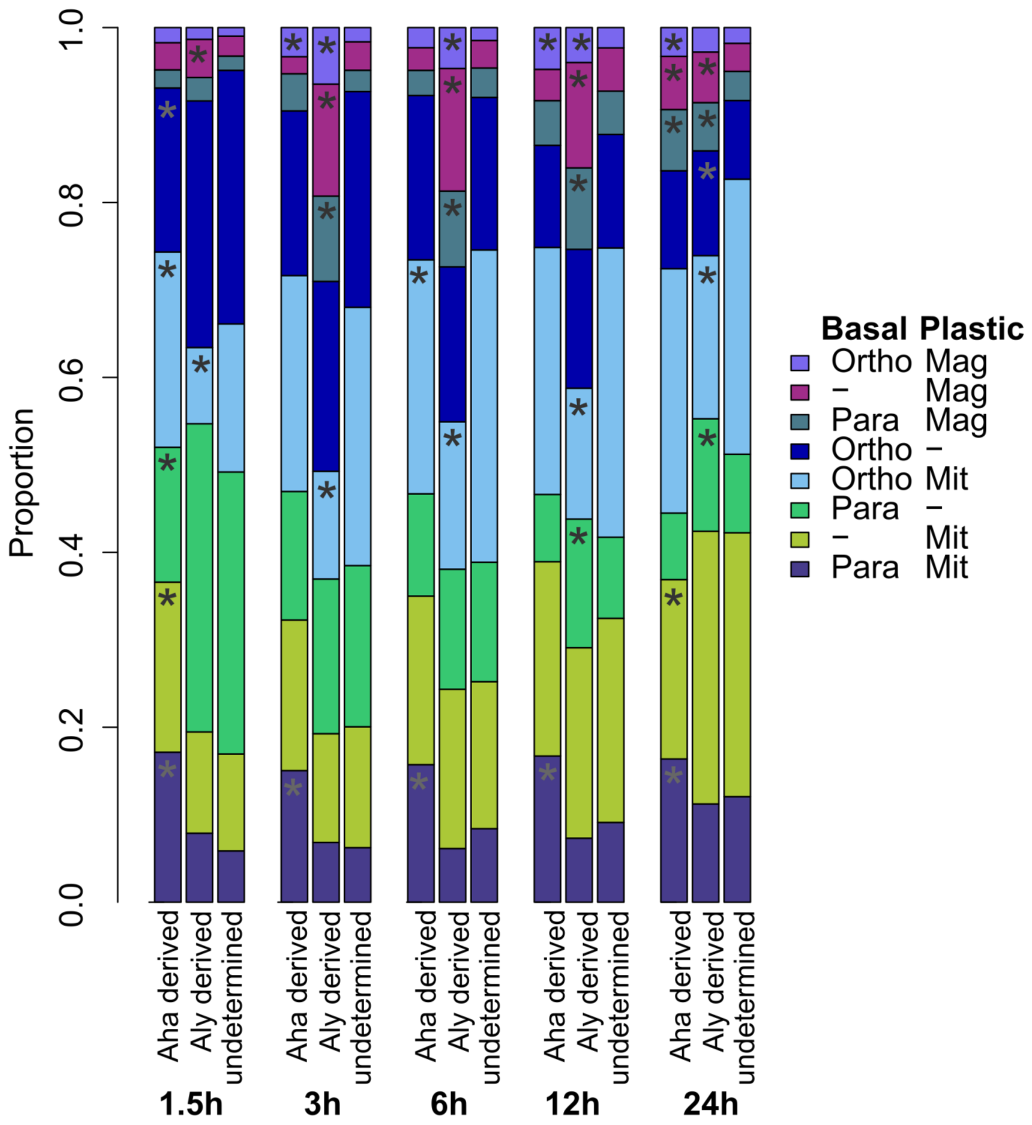

Supplementary Figure 8. **The pattern reported in Figure 4C is also apparent in samples collected after 3h or 12h.**

Ortho-Mag: orthoplasy and magnification, Ortho-Mit: orthoplasy and mitigation, Para-mit: paraplasy and mitigation, Para-Mag: paraplasy and magnification, Only-Mag: magnification only, Only-mit: Mitigation only, Only-Ortho: Orthoplasy only, Only-Para: Paraplasy only.

The proportion of derived and undetermined *cis*-regulatory modifications observed after 6h within each class of genes combining basal and/or plastic expression changes is similar after 3h or 12h of desiccation stress, at the time points where the largest numbers of genes are observed that show a magnified response to stress. Aha - *A. halleri,* Aly– *A. lyrata*.

"*": Significantly increased or decreased number of derived compared to undetermined changes in *A. halleri* or *A. lyrata* at each time point.

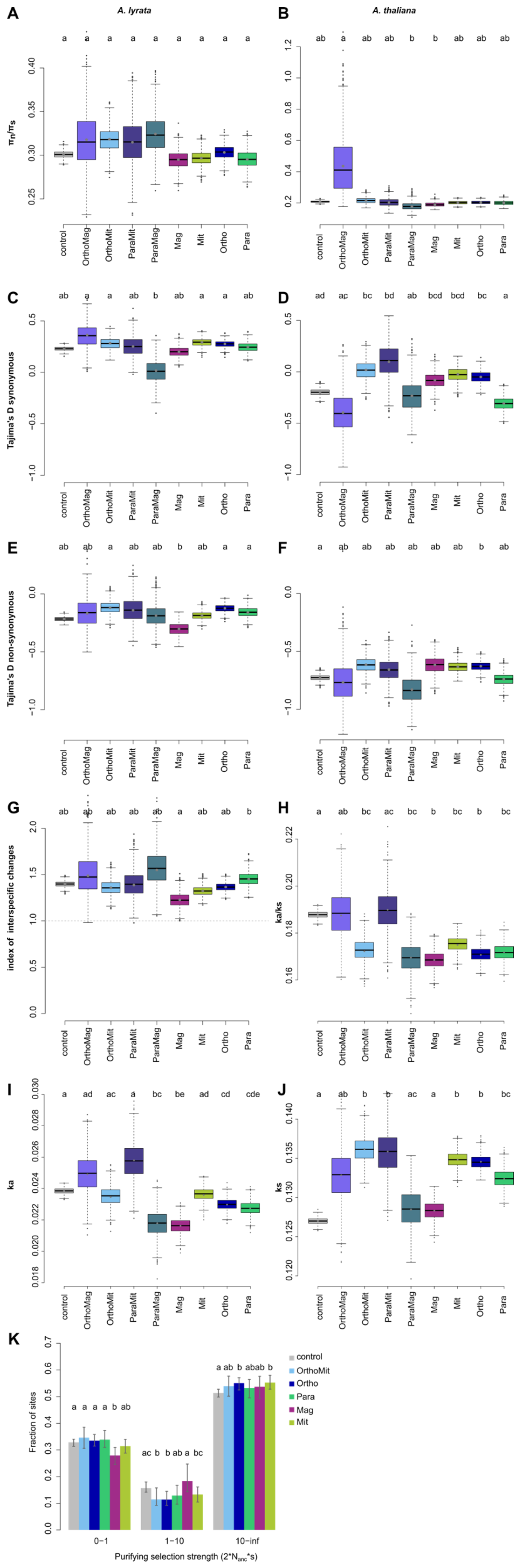

Supplementary Figure 9: **Summary statistics for the 8 plasticity groups and the control for *A. lyrata* (A,C,E) and *A. thaliana* (B,D,F).** Significance between the groups was estimated based on pairwise comparison of 1000 bootstrap replicates, by using the union of the bootstrap values to calculate what proportion of the differences between the groups significantly differed from 0, multiplied by 2, to account for the fact that the test is one-sided. Box and whiskers depict the 75th and 95th interquantile ranges, respectively, dots show outliers. No shared letters mean that the groups are different with a significance cutoff at $p < 0.05$. Pvalues of all pairwise comparisons can be found in Supplementary data 7.

A) and B) pi_n/pi_s, C and D) Tajima's D for synonymous sites, E and F) Tajima's D for non-synonymous sites. G) index of interspecific change for nonsynonymous sites ((A. lyrata +2) / ((A. thaliana +2)), H) ka/ks between *A. thaliana* and *A. lyrata,* I) ka and J) ks.

The plasticity classes OrthoMag, ParaMit and ParaMag were removed from analysis due to the low number of genes and the high variance between the bootstrap in these categories.

K) binned DFE for the control and 5 of the plasticity classes. 95% confidence intervals around the observed mean are based on 200 bootstrap replicates and depicted by error bars. P-values can be found in Supplementary Table 9.

No shared letters between plasticity groups indicates a significant difference between the groups.

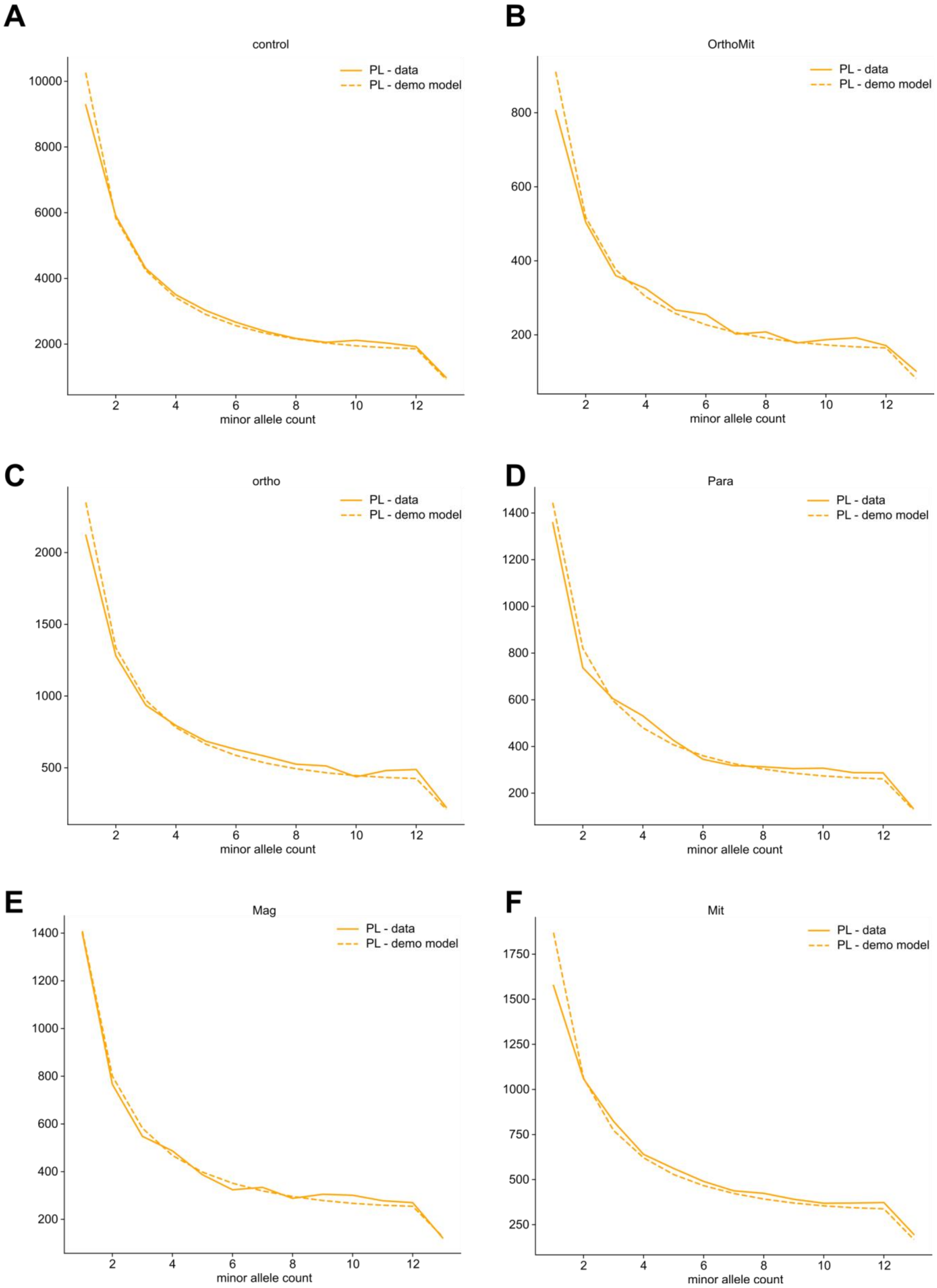

Supplementary Figure 10. Folded site frequency spectra for synonymous sites based on the data (solid line) and based on the expected number of sites of the demographic model (dashed line) for 5 sufficiently large gene sets, that grouped genes by their mode of plasticity evolution, as well as for the group of control genes, which had the same expression levels at all time points in all three species. A Genes with no change in response to stress (Control), B Genes with orthoplastic change in basal gene expression and mitigated response to stress (Ortho-Mit), C Genes with orthoplastic basal gene expression change (ortho), D Genes with paraplastic basal gene expression change (Para), E Genes with magnified response to stress (Mag), F genes with mitigated response to stress (Mit).

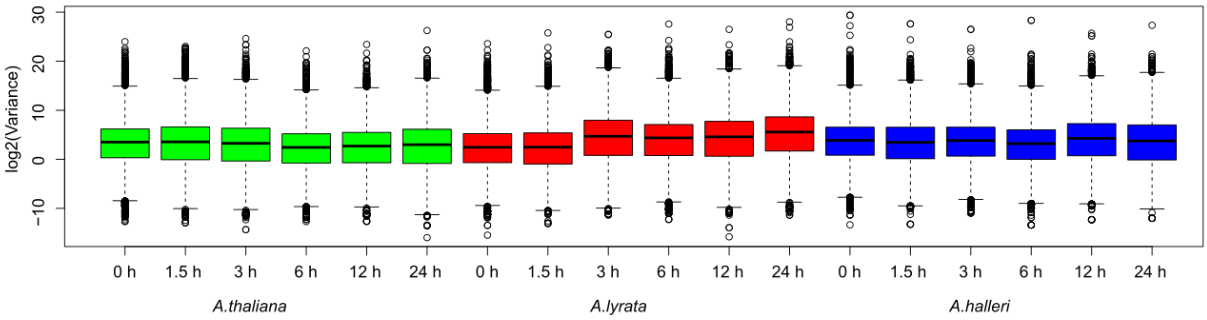

Supplementary Figure 11. **The variance of gene expression among biological replicates**. The variance of the expression level in the samples does not increase with the duration of exposure to the stress. The variance of each gene, in each species and at each time point, was calculated based on the four independent biological replicates of the experiment. Box and whiskers depict the 75th and 95th interquantile ranges, respectively; dots show outliers.

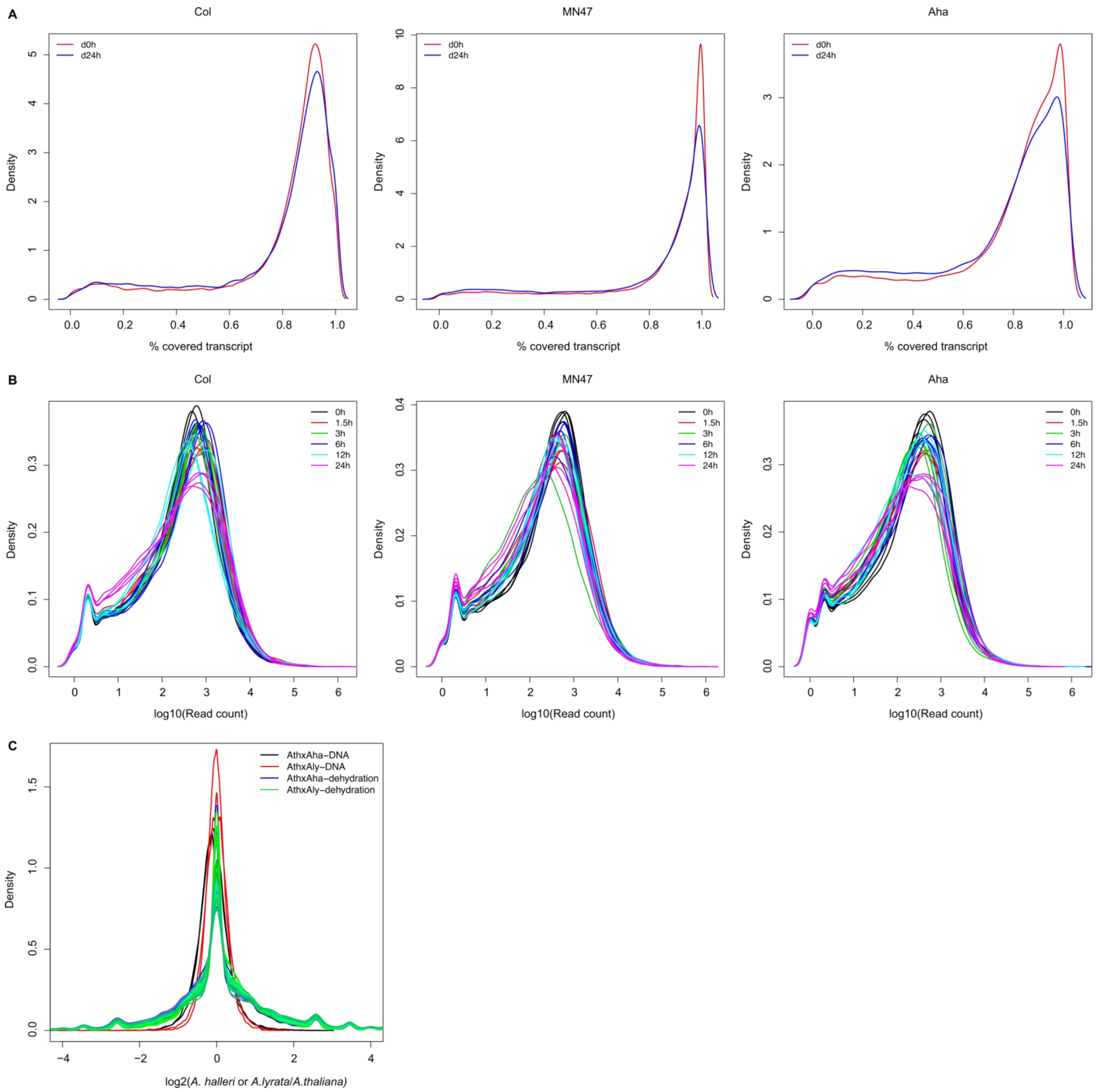

Supplementary Figure 12. **Data quality is comparable across samples.** (A) The proportion of the covered transcript at time point 0 hours (red) and time point 24 hours (blue) for the three species. (B) The distribution of the log10 read count at all time points (0 hours – 24 hours) for the three species and (C) the distribution of the log2 allele ratio in the F1s after remapping and low divergent region filter for the genomes (AthxAha – black; AthxAly – red) and the transcriptomes (AthxAha – blue; AthxAly – green).

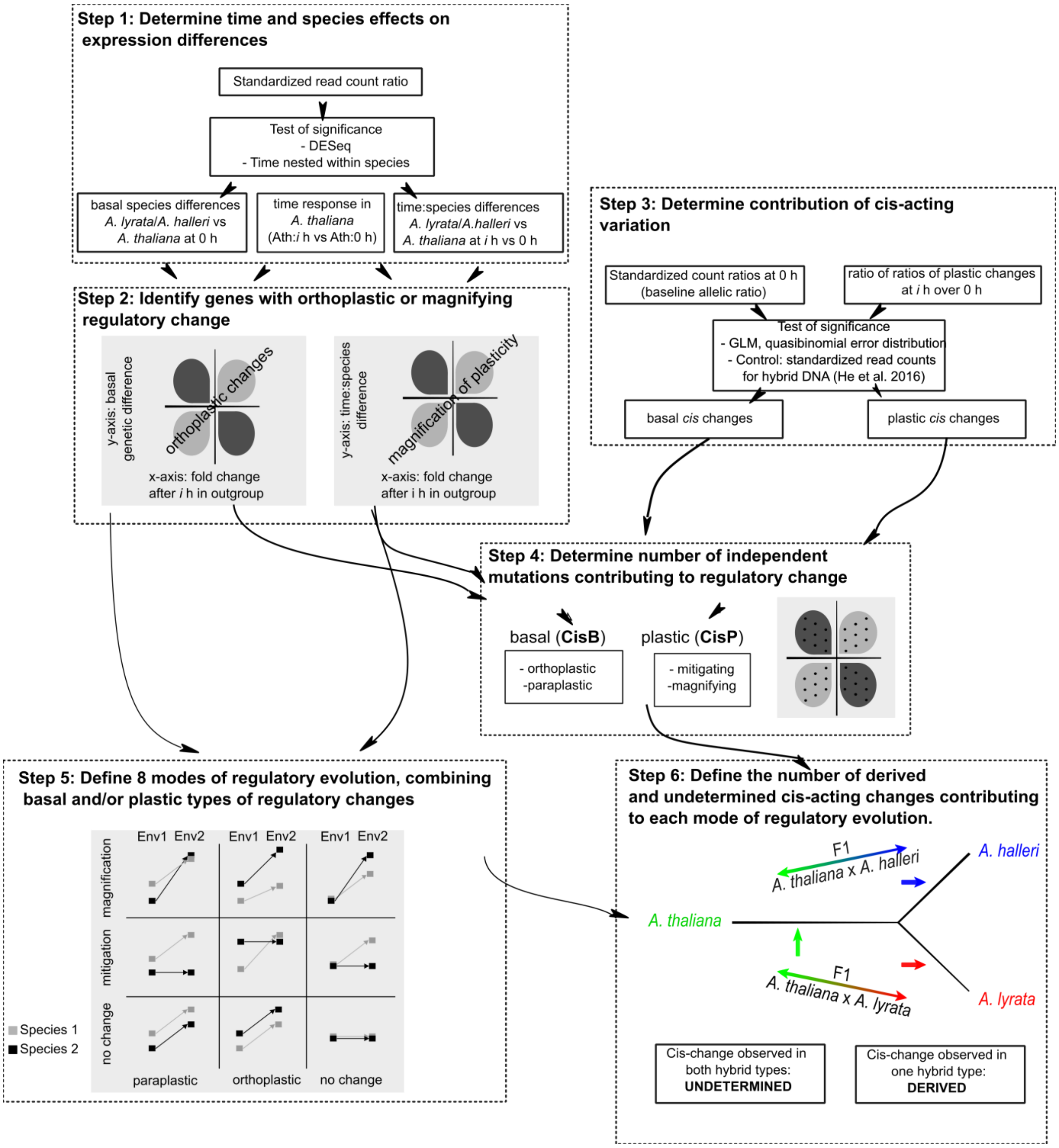

Supplementary Figure 13. Overview of the analytical pipeline to determine basal and plastic expression changes (step 1, step 2) and infer the number of independent *cis*-changes (Step 3, Step 4). Step 5: integration of analyses describing basal and plastic changes in gene regulation in *A. lyrata* and *A. halleri* to define 8 modes of plasticity evolution as well as a group of genes that do not show any differences. Step 6: inferring the derived state of the *cis*-changes observed.

Supplementary Table 1: Percentage of differentially expressed genes that overlap with the differentially expressed genes in Bouzid et al. (2019) for A. halleri and A. lyrata. The study included three different time points, 60% soil moisture (before the stress starts), 20% soil moisture (dehydration stress) and recovery (time point after rewatering). The percentage of up and down regulated in this study is in the top row. Significant overlap between the studies was tested using a hypergeometric test.

		A. halleri 1 vs 0 h	A. halleri 3 vs 0 h	A. halleri 6 vs 0 h	A. halleri 12 vs 0 h	A. halleri 24 vs 0 h
		22% up, 19% down	27% up, 24.9% down	26.5% up, 24.7% down	32.6% up, 30.8% down	35% up, 34.8% down
A. halleri 20 % vs 60	Up (127 ATG	33%	37%	44%	57%	63.7%
% soil moisture	genes)	p= 0.001815975	p = 0.003911163	p= 6.16E-06	p= 2.48E-09	p=9.60E-12
	Down (385 ATG	35%	42.4%	54%	61%	64%
	genes)	p=1.23E-14	p=6.33E-11	p=7.49E-37	p=2.87E-36	p=4.56E-34

		A. halleri 1 vs 0 h	A. halleri 3 vs 0 h	A. halleri 6 vs 0 h	A. halleri 12 vs 0 h	A. halleri 24 vs 0 h
		22% up, 19% down	27% up, 24.9% down	26.5% up, 24.7% down	32.6% up, 30.8% down	35% up, 34.8% down
A. halleri recovery	Up (6 ATG	0	0	0	0	0
vs 60 % soil	genes)	n.s.	n.s.	n.s.	n.s.	n.s.
	Down (7 ATG	28%	42.8%	28%	14%	14%
	genes)	n.s.	p= 0.03514445	n.s.	n.s.	n.s.

		A. lyrata 1 vs 0 h	A. lyrata 3 vs 0 h	A. lyrata 6 vs 0 h	A. lyrata 12 vs 0 h	A. lyrata 24 vs 0 h
		21.8% up; 22.5% down	32% up, 29% down	32.8% up, 30.9% down	35% up, 32% down	37% up, 31% down
A. lyrata 20 % vs 60	Up (15 ATG	40%	46%	53%	53%	53%
% soil moisture	genes)	p= 0.0288373	n.s.	p= 0.02819508	p= 4.51E-02	n.s.
	Down (37 ATG	18.9%	13.5%	13.5%	18.9%	16.2%
	genes)	n.s.	n.s.	n.s.	n.s.	n.s.

		A. lyrata 1 vs 0 h	A. lyrata 3 vs 0 h	A. lyrata 6 vs 0 h	A. lyrata 12 vs 0 h	A. lyrata 24 vs 0 h
		21.8% up; 22.5% down	32% up, 29% down	32.8% up, 30.9% down	35% up, 32% down	37% up, 31% down
A. lyrata recovery vs	Up (61 ATG	32.7%	29.5%	34.4%	34.4%	36%
60 % soil moisture	genes)	p= 0.01640409	n.s.	p= 5.94947E-40	n.s.	n.s.
	Down (90 ATG	27.7%	44.4%	44.4%	61%	51%
	genes)	n.s.	p= 8.54E-04	p= 2.25E-03	p= 4.49E-09	p= 2.26E-05

Supplementary Table 2: Proportion of stress-response genes in each species whose basal expression or slope (plastic difference) differs from another species. For each species, we counted the number of genes showing stress response comparing expression at tpx with tp0 (FDR 0.05), and then calculated how many of them showed an basal gene expression difference between species at tp0 (species1-tp0/species2-tp0) or a species differences in the slope of the plastic reaction between tpx and tp0 ((species1/species2-tpx)/(species1/species2-tp0)) at FDR 0.05.

	Aha				Aly				Ath			
	basal difference		plastic difference		basal difference		plastic difference		basal difference		plastic difference	
Time point	Aha vs Ath	Aha vs Aly	Aha vs Ath	Aha vs Aly	Aly vs Ath	Aly vs Aha	Aly vs Ath	Aly vs Aha	Ath vs Aha	Ath vs Aly	Ath vs Aha	Ath vs Aly
1.5h	56.59%	52.75%	30.27%	11.36%	40.05%	52.68%	12.78%	13.62%	55.34%	38.98%	30.02%	13.08%
3h	55.88%	52.79%	34.75%	17.74%	39.86%	53.42%	30.78%	20.93%	57.38%	41.27%	34.57%	28.51%
6h	56.86%	53.42%	35.46%	22.74%	39.47%	53.21%	33.58%	27.05%	57.86%	40.73%	37.48%	32.31%
12h	56.32%	52.80%	45.71%	26.52%	40.20%	53.93%	34.14%	28.59%	57.35%	40.27%	48.22%	34.08%
24h	56.35%	52.97%	45.06%	31.43%	39.79%	53.60%	36.61%	30.55%	56.64%	39.32%	47.09%	38.82%

Supplementary Table 3: The Pearson's correlation (r) of log ratio of allelic expression difference between hybrid and parents computed separately for genes with a significant cis change and a significant difference between parents (cis), a significant difference between parents only (trans only) and a significant cis change in hybrid only (weak cis). At tp 0, the allelic difference is the ratio of expression difference in alleles between species (species1-tp0 / species2-tp0). At tpx, the allelic difference is the ratio of ratio of allelic difference of species between tpx and tp0 ((species1/spcies2-tpx) / (speices1/species2-tp0)). *Cis* means both of significant difference in the hybrid and parents with the same direction of changes. *Trans* means that there is no significant cis change in the hybrids. Compensatory means only differences between hybrid alleles not in parents, but they are still correlated to the parents. All correlations are highly significant p < 2.2e-16.

	Aha			Aly		
Time point	cis	trans only	weak cis	cis	trans only	weak cis
0h	0.814	0.234	0.299	0.854	0.467	0.514
1.5h	0.851	0.244	0.483	0.952	0.478	0.722
3h	0.848	0.287	0.575	0.916	0.623	0.657
6h	0.868	0.297	0.563	0.911	0.636	0.631
12h	0.836	0.289	0.452	0.925	0.653	0.683
24h	0.849	0.324	0.552	0.921	0.551	0.683

Supplementary Table 4. GO enrichment of plastic genes in each mode of plasticity evolution in *A. halleri* and *A. lyrata* (p<10-5). The mode of plasticity is determined by the combination of basal (orthoplasy / Paraplasy) and plastic (Magnification / Mitigation) changes. GO analysis was done using TopGO with the elim method. Enrichment were computed against the reference ensemble of all the expressed orthologous genes of three species. The GO term with less than 2 genes in the column "significant" were removed.

GO.ID	Term	Annotated	Significant	Expected	resultFisher	basal	platic	species
GO:0009220	pyrimidine ribonucleotide biosynthetic p...	111	9	0.87	0.0000002	Orthoplasy	Magnification	A.lyrata
GO:0006270	DNA replication initiation	55	17	0.78	8E-19	-	Magnification	A.halleri
GO:0006275	regulation of DNA replication	115	19	1.64	2.3E-15	-	Magnification	A.halleri
GO:0008283	cell proliferation	208	23	2.97	1.9E-14	-	Magnification	A.halleri
GO:0051567	histone H3-K9 methylation	152	19	2.17	4.4E-13	-	Magnification	A.halleri
GO:0006306	DNA methylation	143	16	2.04	1.9E-10	-	Magnification	A.halleri
GO:0009909	regulation of flower development	284	21	4.05	6.2E-10	-	Magnification	A.halleri
GO:0034968	histone lysine methylation	193	27	2.75	4.1E-08	-	Magnification	A.halleri
GO:0016458	gene silencing	298	20	4.25	8.6E-08	-	Magnification	A.halleri
GO:0016572	histone phosphorylation	46	8	0.66	2.4E-07	-	Magnification	A.halleri
GO:0010389	regulation of G2/M transition of mitotic...	59	8	0.84	0.0000017	-	Magnification	A.halleri
GO:0010075	regulation of meristem growth	136	11	1.94	0.0000038	-	Magnification	A.halleri
GO:0000911	cytokinesis by cell plate formation	169	12	2.41	0.0000053	-	Magnification	A.halleri
GO:0032508	DNA duplex unwinding	10	4	0.14	0.0000078	-	Magnification	A.halleri
GO:0008283	cell proliferation	208	58	9.33	1E-30	-	Magnification	A.lyrata
GO:0000911	cytokinesis by cell plate formation	169	46	7.58	5.8E-24	-	Magnification	A.lyrata
GO:0006275	regulation of DNA replication	115	38	5.16	2.5E-23	-	Magnification	A.lyrata
GO:0006270	DNA replication initiation	55	25	2.47	1E-19	-	Magnification	A.lyrata
GO:0051567	histone H3-K9 methylation	152	39	6.82	1.8E-19	-	Magnification	A.lyrata
GO:0006306	DNA methylation	143	34	6.41	4.9E-16	-	Magnification	A.lyrata
GO:0010389	regulation of G2/M transition of mitotic...	59	22	2.65	2.7E-15	-	Magnification	A.lyrata
GO:0010103	stomatal complex morphogenesis	122	29	5.47	7.4E-14	-	Magnification	A.lyrata
GO:0016572	histone phosphorylation	46	17	2.06	4.9E-12	-	Magnification	A.lyrata
GO:0006261	DNA-dependent DNA replication	204	53	9.15	3.6E-11	-	Magnification	A.lyrata
GO:0009909	regulation of flower development	284	39	12.74	3.7E-10	-	Magnification	A.lyrata
GO:0019288	isopentenyl diphosphate biosynthetic pro...	188	29	8.43	4.9E-09	-	Magnification	A.lyrata
GO:0007020	microtubule nucleation	52	14	2.33	4.1E-08	-	Magnification	A.lyrata
GO:0009902	chloroplast relocation	89	18	3.99	6.3E-08	-	Magnification	A.lyrata
GO:0051225	spindle assembly	40	12	1.79	0.0000001	-	Magnification	A.lyrata
GO:0006364	rRNA processing	215	29	9.64	0.0000001	-	Magnification	A.lyrata
GO:0010075	regulation of meristem growth	136	22	6.1	1.5E-07	-	Magnification	A.lyrata
GO:0009965	leaf morphogenesis	176	25	7.89	2.9E-07	-	Magnification	A.lyrata
GO:0000226	microtubule cytoskeleton organization	200	44	8.97	9.5E-07	-	Magnification	A.lyrata
GO:0006084	acetyl-CoA metabolic process	66	14	2.96	0.000001	-	Magnification	A.lyrata
GO:0007169	transmembrane receptor protein tyrosine ...	87	16	3.9	0.0000013	-	Magnification	A.lyrata
GO:0032508	DNA duplex unwinding	10	6	0.45	0.0000014	-	Magnification	A.lyrata
GO:0009220	pyrimidine ribonucleotide biosynthetic p...	111	18	4.98	0.000002	-	Magnification	A.lyrata
GO:0006412	translation	304	122	34.96	1E-30	Orthoplasy	-	A.halleri
GO:0019288	isopentenyl diphosphate biosynthetic pro...	188	73	21.62	1.9E-22	Orthoplasy	-	A.halleri
GO:0006364	rRNA processing	215	79	24.72	2E-22	Orthoplasy	-	A.halleri
GO:0010027	thylakoid membrane organization	162	54	18.63	1.1E-13	Orthoplasy	-	A.halleri
GO:0001510	RNA methylation	116	43	13.34	6.1E-13	Orthoplasy	-	A.halleri
GO:0015995	chlorophyll biosynthetic process	107	40	12.3	2.9E-12	Orthoplasy	-	A.halleri
GO:0042254	ribosome biogenesis	262	101	30.13	4.3E-10	Orthoplasy	-	A.halleri
GO:0010207	photosystem II assembly	139	42	15.98	1.9E-09	Orthoplasy	-	A.halleri
GO:0016226	iron-sulfur cluster assembly	83	30	9.54	3.8E-09	Orthoplasy	-	A.halleri
GO:0009658	chloroplast organization	202	65	23.23	4E-09	Orthoplasy	-	A.halleri

GO:0045036	protein targeting to chloroplast	57	24	6.55	4.1E-09	Orthoplasy	-	A.halleri
GO:0016117	carotenoid biosynthetic process	86	30	9.89	1E-08	Orthoplasy	-	A.halleri
GO:0006636	unsaturated fatty acid biosynthetic proc...	55	23	6.32	1E-08	Orthoplasy	-	A.halleri
GO:0019344	cysteine biosynthetic process	158	44	18.17	1.3E-08	Orthoplasy	-	A.halleri
GO:0009657	plastid organization	327	109	37.6	2.6E-08	Orthoplasy	-	A.halleri
GO:0010103	stomatal complex morphogenesis	122	36	14.03	5.4E-08	Orthoplasy	-	A.halleri
GO:0009073	aromatic amino acid family biosynthetic ...	79	27	9.08	8.9E-08	Orthoplasy	-	A.halleri
GO:0009902	chloroplast relocation	89	29	10.23	9.9E-08	Orthoplasy	-	A.halleri
GO:0006399	tRNA metabolic process	92	36	10.58	2.5E-07	Orthoplasy	-	A.halleri
GO:0006098	pentose-phosphate shunt	155	39	17.82	0.0000015	Orthoplasy	-	A.halleri
GO:0009220	pyrimidine ribonucleotide biosynthetic p...	111	31	12.76	0.0000017	Orthoplasy	-	A.halleri
GO:0006655	phosphatidylglycerol biosynthetic proces...	58	20	6.67	0.0000035	Orthoplasy	-	A.halleri
GO:0042274	ribosomal small subunit biogenesis	11	8	1.26	0.0000036	Orthoplasy	-	A.halleri
GO:0006418	tRNA aminoacylation for protein translat...	37	15	4.25	0.000006	Orthoplasy	-	A.halleri
GO:0042793	plastid transcription	66	21	7.59	0.0000086	Orthoplasy	-	A.halleri
GO:0001510	RNA methylation	116	29	9.29	2.2E-08	Orthoplasy	-	A.lyrata
GO:0006412	translation	304	47	24.36	0.0000088	Orthoplasy	-	A.lyrata
GO:0006914	autophagy	56	15	3.27	4.7E-07	Paraplasy	-	A.lyrata
GO:0010200	response to chitin	292	37	17.07	0.000007	Paraplasy	-	A.lyrata

Supplementary Table 5. GO enrichment of plastic genes with derived or undetermined cis changes in each mode of plasticity evolution in *A. halleri* and *A. lyrata*. The mode of plasticity evolution is determined by the combination of basal (**Ortho**plasy / **Para**plasy) and plastic (**Mag**nification / **Mit**igation) changes. GO analysis was done using TopGO with the elim method. Enrichment were computed against the reference ensemble of all the expressed orthologous genes of three species. The GO term with less than 2 genes in the column "significant" were removed.

GO.ID	Term	Annotated	Significant	Expected	resultFisher	basal	plastic	cis
GO:0001510	RNA methylation	116	3	0.21	0.0011	Ortho	Mag	AH der
GO:0016572	histone phosphorylation	46	2	0.08	0.0031	Ortho	Mag	AH der
GO:0006333	chromatin assembly or disassembly	50	2	0.09	0.0036	Ortho	Mag	AH der
GO:0006636	unsaturated fatty acid biosynthetic proc...	55	2	0.1	0.0044	Ortho	Mag	AH der
GO:0008283	cell proliferation	208	3	0.38	0.006	Ortho	Mag	AH der
GO:0009220	pyrimidine ribonucleotide biosynthetic p...	111	5	0.35	2.30E-05	Ortho	Mag	AL der
GO:0008654	phospholipid biosynthetic process	306	5	0.96	2.50E-03	Ortho	Mag	AL der
GO:0046148	pigment biosynthetic process	219	4	0.69	4.70E-03	Ortho	Mag	AL der
GO:0010383	cell wall polysaccharide metabolic proce...	177	3	0.26	0.0021	-	Mag	AH der
GO:0009736	cytokinin-activated signaling pathway	59	2	0.09	0.0034	-	Mag	AH der
GO:0042546	cell wall biogenesis	268	3	0.4	0.0068	-	Mag	AH der
GO:0010207	photosystem II assembly	139	10	1.43	1.60E-06	-	Mag	AL der
GO:0006364	rRNA processing	215	12	2.22	2.10E-06	-	Mag	AL der
GO:0016998	cell wall macromolecule catabolic proces...	10	4	0.1	2.20E-06	-	Mag	AL der
GO:0010103	stomatal complex morphogenesis	122	9	1.26	4.50E-06	-	Mag	AL der
GO:0035304	regulation of protein dephosphorylation	114	8	1.17	2.20E-05	-	Mag	AL der
GO:0019288	isopentenyl diphosphate biosynthetic pro...	188	10	1.94	2.40E-05	-	Mag	AL der
GO:0009965	leaf morphogenesis	176	9	1.81	8.40E-05	-	Mag	AL der
GO:0043085	positive regulation of catalytic activit...	104	7	1.07	9.70E-05	-	Mag	AL der
GO:0019344	cysteine biosynthetic process	158	8	1.63	2.20E-04	-	Mag	AL der
GO:0045893	positive regulation of transcription, DN...	356	12	3.67	3.00E-04	-	Mag	AL der
GO:0040034	regulation of development, heterochronic	32	2	0.06	0.0015	Para	Mag	AH der
GO:0048767	root hair elongation	143	3	0.26	0.0021	Para	Mag	AH der
GO:0009059	macromolecule biosynthetic process	2572	11	4.66	0.0026	Para	Mag	AH der
GO:0034968	histone lysine methylation	193	5	1.08	4.50E-03	Para	Mag	AL der
GO:0007000	nucleolus organization	19	2	0.11	5.00E-03	Para	Mag	AL der
GO:0009072	aromatic amino acid family metabolic pro...	167	11	3.14	0.00031	Ortho	Mit	AH der
GO:1901606	alpha-amino acid catabolic process	110	8	2.07	0.00109	Ortho	Mit	AH der
GO:0010363	regulation of plant-type hypersensitive ...	275	13	5.17	0.0021	Ortho	Mit	AH der
GO:0006612	protein targeting to membrane	276	13	5.19	0.00217	Ortho	Mit	AH der

GO:0009651	response to salt stress	571	21	10.73	0.00254	Ortho	Mit	AH der
GO:0006972	hyperosmotic response	193	10	3.63	0.00353	Ortho	Mit	AH der
GO:0010033	response to organic substance	1978	53	37.18	0.00386	Ortho	Mit	AH der
GO:0009963	positive regulation of flavonoid biosynt...	80	6	1.5	0.0039	Ortho	Mit	AH der
GO:0046474	glycerophospholipid biosynthetic process	137	8	2.57	0.00432	Ortho	Mit	AH der
GO:0046885	regulation of hormone biosynthetic proce...	18	3	0.34	0.00434	Ortho	Mit	AH der
GO:0009593	detection of chemical stimulus	13	3	0.15	0.00038	Ortho	Mit	AL der
GO:0006984	ER-nucleus signaling pathway	12	3	0.15	0.00041	Ortho	-	AH der
GO:0006364	rRNA processing	215	10	2.73	0.00041	Ortho	-	AH der
GO:0019288	isopentenyl diphosphate biosynthetic pro...	188	9	2.39	0.00065	Ortho	-	AH der
GO:0045036	protein targeting to chloroplast	57	5	0.72	0.00076	Ortho	-	AH der
GO:0006790	sulfur compound metabolic process	534	16	6.78	0.00123	Ortho	-	AH der
GO:1901566	organonitrogen compound biosynthetic pro...	1464	35	18.59	0.00161	Ortho	-	AH der
GO:0015931	nucleobase-containing compound transport	108	6	1.37	0.00251	Ortho	-	AH der
GO:0006006	glucose metabolic process	198	8	2.51	0.00375	Ortho	-	AH der
GO:0009658	chloroplast organization	202	8	2.56	0.00423	Ortho	-	AH der
GO:0015994	chlorophyll metabolic process	161	7	2.04	0.00444	Ortho	-	AH der
GO:0042138	meiotic DNA double-strand break formatio...	62	4	0.5	0.0015	Para	-	AH der
GO:0006312	mitotic recombination	45	3	0.36	0.0055	Para	-	AH der
GO:0009560	embryo sac egg cell differentiation	111	6	1.03	0.00059	Para	-	AL der
GO:0001101	response to acid chemical	1084	26	15.28	0.005	-	Mit	AH der
GO:0014070	response to organic cyclic compound	516	15	7.27	0.0063	-	Mit	AH der
GO:0006766	vitamin metabolic process	72	6	0.91	0.0003	-	Mit	AL der
GO:0009269	response to desiccation	22	3	0.28	0.0026	-	Mit	AL der
GO:0009845	seed germination	193	8	2.45	0.0032	-	Mit	AL der
GO:0009106	lipoate metabolic process	27	3	0.34	0.0047	-	Mit	AL der
GO:0009072	aromatic amino acid family metabolic pro...	167	7	2.12	0.0054	-	Mit	AL der
GO:0031667	response to nutrient levels	218	8	2.77	0.0066	-	Mit	AL der
GO:0009638	phototropism	10	2	0.13	0.0067	-	Mit	AL der

Supplementary Table 6: The proportion of mapped RNAseq reads for the three species and the F1 hybrids with different reference genomes. As the draft genome of *A. halleri* v1.1 in Phytozome 12.1 can not cover the whole transcriptome of *A. halleri*, we used the *A. lyrata* genome as the reference for *A. halleri* and its hybrid. We further generated an *A. halleri* pseudo genome (see method) and used it as a reference for *A. halleri* and its hybrid. The pseudogenome increases the mapping efficiency.

Genotype	Reference	0h	1.5h	3h	6h	12h	24h
Ath	A. thaliana	92%	92%	92%	92%	91%	90%
Aly	A.lyrata	90%	90%	90%	89%	88%	87%
Aha	A.lyrata	70%	70%	70%	70%	68%	67%
Aha	pseudo gen	90%	90%	90%	90%	89%	88%
AthXAly	thxAly genom	90%	90%	90%	90%	89%	88%
AthxAha	thxAly genom	80%	80%	80%	80%	79%	78%
AthxAha	ha pseudoge	89%	89%	89%	89%	88%	88%

Supplementary Table 7: The gene number and contribution of basal/plastic cis changes for each mode of plasticity evolution. CisB is basal cis change, cisP is plastic cis change, and cisBP is both of basal and plastic change.

	A.halleri				A.lyrata			
Mode of plasticity evolution	Genes	cisB	cisP	cisBP	Genes	cisB	cisP	cisBP
orthoplasy - magnification	119	19	35	7	118	37	25	13
magnification	209	-	74	-	629	-	222	-
paraplasy - magnification	109	19	20	29	208	45	45	56
orthoplasy - mitigation	1205	99	329	248	679	60	195	230
orthoplasy	1662	393	-	-	1178	475	-	-
paraplasy	975	252	-	-	860	315	-	-
paraplasy - mitigation	564	64	185	49	219	42	58	46
mitigation	796	-	406	-	735	-	442	-

Supplementary Table 8 List of plant populations and their geographical distribution of the dehydration experiment in Supplementary Figure 1.

Species	Population	Accession	Location	Latitude	Longitude
A. halleri	Laut	Laut3	Germany	49.23	14.04
A. halleri	Wall	Wall7	Germany	49.73	12.91
A. halleri	Kowa	Kowa4	Poland	50.63	16.08
A. halleri	Bara	Bara4	Romania	45.55	22.9
A. halleri	Nisu	Nisu6	Romania	46.24	22.55
A. halleri	lita	Lita6	Slovenia	46.76	15.79
A. halleri	Lobn	Lobn5	Slovenia	46.69	16.16
A. halleri	Noss	Noss5	Italy	44.28	14.96
A. halleri	Pais	Pais9	Italy	46.05	10.24
A. halleri	hal4.11	hal4.11	Italy	45.86	9.84
A. lyrata	SB	SB12	Germany	51.31	10.55
A. lyrata	LF	LF10	Austria	47.59	15.36
A. lyrata	LF	LF2	Austria	47.59	15.36
A. lyrata	NT	NT12	Germany	49.31	11.32
A. lyrata	VH	Vosshütte	Austria	47.58	16.1
A. lyrata		Tannenberg1			
A. lyrata	VOS	VOS	Austria	57.58	16. Okt
A. lyrata	Plech	Plech.Rock79b	Germany	49.37	11.3
A. lyrata	Plech	Plech61.2a	Germany	49.37	11.3
A. lyrata	Plech	Plech91.4a	Germany	49.37	11.3
A. lyrata	Plech	Plech92.2a	Germany	49.37	11.3
A. lyrata	Plech	PlechC3	Germany	49.37	11.3
A. lyrata	HAS	HAS.120	Germany	49.47	11.25
A. lyrata	HAS	HAS122c	Germany	49.47	11.25
A. lyrata	HAS	HAS166b	Germany	49.47	11.25
A. lyrata	MN47	MN47	US	44.31	-85.6
A. lyrata	Sky	Sky	Scotland	57.54	-6.17
A. thaliana	IP-Ara-4	IP.Ara4	Spain	41.7	-3.68

A. thaliana	IP-Cmo-3	IP.Cmo3	Spain	40.05	-4.65
A. thaliana	IP-Hoy-0	IP.Hoy0	Spain	40.4	-5
A. thaliana	IP-lab-7	IP.Lab7	Spain	40.87	-4.5
A. thaliana	Amu-0	Amu0	Spain	42.35	-3.03
A. thaliana	Coy-0	Coy0	Spain	40.44	-4.27
A. thaliana	Gud-3	Gud3	Spain	40.65	-4.11
A. thaliana	Hec-0	Hec0	Spain	42.86	-0.7
A. thaliana	Pdl-0	Pdl0	Spain	43.02	-5.6
A. thaliana	Prd-0	Prd0	Spain	41.14	-3.68
A. thaliana	Som-0	Som0	Spain	41.14	-3.58
A. thaliana	Urd-1	Urd1	Spain	42.27	-2.98
A. thaliana	Val-0	Val0	Spain	42.31	-3.1
A. thaliana	Col.FRI	Col.FRI	NA	NA	NA

Supplementary Table 9: Pvalues for pairwise comparisons of the bins of the different plasticity categories and the control genes for the DFE in Supplementary Figure 9.

bin 0-1	ortho-mit	ortho	para	mag	mit
control	0.43	0.61	0.61	**0.01**	0.41
ortho-mit	NA	0.76	0.82	**0.01**	0.25
ortho		NA	0.98	**0.01**	0.25
para			NA	**0.01**	0.27
mag				NA	0.09
bin 1-10	ortho-mit	ortho	para	mag	mit
control	**0.03**	**0.01**	0.17	0.32	0.15
ortho-mit	NA	0.98	0.7	**0.04**	0.51
ortho		NA	0.55	**0.01**	0.38
para			NA	0.09	0.81
mag				NA	**0.03**
bin 10-inf	ortho-mit	ortho	para	mag	mit
control	0.18	**0.01**	0.26	0.37	**0.02**
ortho-mit	NA	0.5	0.95	0.99	0.55
ortho		NA	0.39	0.62	0.93
para			NA	0.97	0.45
mag				NA	0.48
