# Supplementary material for: Cis-regulatory evolution spotlights species differences in the adaptive potential of gene expression plasticity
Source: Nat Commun. 2021 Jun 7;12:3376. doi: 10.1038/s41467-021-23558-2 (PMC8184852; doi:10.1038/s41467-021-23558-2)
Supplement: Supplementary file 3 — Descriptions of Additional Supplementary Files [file 41467_2021_23558_MOESM3_ESM.pdf]

Descriptions of Additional Supplementary Files

Supplementary data 1
Description: Weight loss during the desiccation experiment. The aerial part of the plants was cut at the base of the roots to mimic a rupture of the water column, as occurs when the plant wilts, and deposited on absorbent paper for 0, 1.5, 3, 6, 12 and 24 hours in the growth chamber under the same growth conditions. Plants were weighed before and after the stress prior to flash freezing for RNA extraction.

Supplementary data 2
Description: Survival after a desiccation duration of 6h, 12h or 24h . Plants were removed from the soil and repotted after 0h, 6h, 12h and 24h of dehydration. Survival was measured after 18 days. This experiment included a representative set of 10, 17 and 14 genotypes of A. halleri, A. lyrata and A. thaliana, respectively.

Supplementary data 3
Description: List of plastic genes in A. thaliana (Ath) after 6 hours of desiccation and their basal change or plastic response in A. halleri (Aha) and A. lyrata (Aly). Plastic genes were selected by comparing time point 6h with 0h in A. thaliana with an FDR threshold of 0.05. The mean expression levels before and after the stress are shown as the mean FPKM of 4 replicates. For each plastic gene in A. thaliana, log2 fold change (log2FoldChange), adjusted p value (padj) and direction of regulation (Up or Down) is shown. For each A. halleri and A. lyrata, basal changes and plastic response compared to A. thaliana is shown with log2 fold change (log2FoldChange), adjusted p value (padj) and the information in which plastic group the gene is located (basal or plastic). The basal change was calculated using the difference at time point 0h (Aha-0h or Aly-0h vs Ath-0h; “Ortho” is orthoplasy, “Para” is paraplasy, and “-” is no plasticity). The plastic response was calculated by the difference in the response to the stress at 6h (Aha/Ath-6h vs Aha/Ath-0h or Aly/Ath-6h vs Aly/Ath-0h; “Mag” is Magnification, “Mit” is Mitigation, and “-” is no plasticity).

Supplementary data 4
Description: List of plastic genes in A. thaliana (Ath) after 1.5 hours of desiccation and their basal change or plastic response in A. halleri (Aha) and A. lyrata (Aly). Plastic genes were selected by comparing time point 1.5h with 0h in A. thaliana with an FDR threshold of 0.05. The mean expression levels before and after the stress are shown as the mean FPKM of 4 replicates. For each plastic gene in A. thaliana, log2 fold change (log2FoldChange), adjusted p value (padj) and direction of regulation (Up or Down) is shown. For each A. halleri and A. lyrata, basal changes and plastic response compared to A. thaliana is shown with log2 fold change (log2FoldChange), adjusted p value (padj) and the information in which plastic group the gene is located (basal or plastic). The basal change was calculated using the difference at time point 0h (Aha-0h or Aly-0h vs Ath-0h; “Ortho” is orthoplasy, “Para” is paraplasy, and “- ” is no plasticity). The plastic response was calculated by the difference in the response to the stress at 1.5h (Aha/Ath-1.5h vs Aha/Ath-0h or Aly/Ath-1.5h vs Aly/Ath-0h; “Mag” is Magnification, “Mit” is Mitigation, and “-” is no plasticity).

Supplementary data 5
Description: List of plastic genes in A. thaliana (Ath) after 3 hours of desiccation and their basal change or plastic response in A. halleri (Aha) and A. lyrata (Aly). Plastic genes were selected by comparing time point 3h with 0h in A. thaliana with an FDR threshold of 0.05. The mean expression levels before and after the stress are shown as the mean FPKM of 4 replicates. For each plastic gene in A. thaliana, log2 fold change (log2FoldChange), adjusted p value (padj) and direction of regulation (Up or Down) is shown. For each A. halleri and A. lyrata, basal changes and plastic response

compared to A. thaliana is shown with log2 fold change (log2FoldChange), adjusted p value (padj) and the information in which plastic group the gene is located (basal or plastic). The basal change was calculated using the difference at time point 0h (Aha-0h or Aly-0h vs Ath-0h; “Ortho” is orthoplasy, “Para” is paraplasy, and “-” is no plasticity). The plastic response was calculated by the difference in the response to the stress at 3h (Aha/Ath-3h vs Aha/Ath-0h or Aly/Ath-3h vs Aly/Ath-0h; “Mag” is Magnification, “Mit” is Mitigation, and “-” is no plasticity).

Supplementary data 6
Description: List of plastic genes in A. thaliana (Ath) after 12 hours of desiccation and their basal change or plastic response in A. halleri (Aha) and A. lyrata (Aly). Plastic genes were selected by comparing time point 12h with 0h in A. thaliana with an FDR threshold of 0.05. The mean expression levels before and after the stress are shown as the mean FPKM of 4 replicates. For each plastic gene in A. thaliana, log2 fold change (log2FoldChange), adjusted p value (padj) and direction of regulation (Up or Down) is shown. For each A. halleri and A. lyrata, basal changes and plastic response compared to A. thaliana is shown with log2 fold change (log2FoldChange), adjusted p value (padj) and the information in which plastic group the gene is located (basal or plastic). The basal change was calculated using the difference at time point 0h (Aha-0h or Aly-0h vs Ath-0h; “Ortho” is orthoplasy, “Para” is paraplasy, and “-” is no plasticity). The plastic response was calculated by the difference in the response to the stress at 12h (Aha/Ath-12h vs Aha/Ath-0h or Aly/Ath-12h vs Aly/Ath-0h; “Mag” is Magnification, “Mit” is Mitigation, and “-” is no plasticity).

Supplementary data 7
Description: List of plastic genes in A. thaliana (Ath) after 24 hours of desiccation and their basal change or plastic response in A. halleri (Aha) and A. lyrata (Aly). Plastic genes were selected by comparing time point 24h with 0h in A. thaliana with an FDR threshold of 0.05. The mean expression levels before and after the stress are shown as the mean FPKM of 4 replicates. For each plastic gene in A. thaliana, log2 fold change (log2FoldChange), adjusted p value (padj) and direction of regulation (Up or Down) is shown. For each A. halleri and A. lyrata, basal changes and plastic response compared to A. thaliana is shown with log2 fold change (log2FoldChange), adjusted p value (padj) and the information in which plastic group the gene is located (basal or plastic). The basal change was calculated using the difference at time point 0h (Aha-0h or Aly-0h vs Ath-0h; “Ortho” is orthoplasy, “Para” is paraplasy, and “-” is no plasticity). The plastic response was calculated by the difference in the response to the stress at 24h (Aha/Ath-24h vs Aha/Ath-0h or Aly/Ath-24h vs Aly/Ath-0h; “Mag” is Magnification, “Mit” is Mitigation, and “-” is no plasticity).

Supplementary data 8
Description: Pvalues for pairwise comparisons of the different plasticity categories and control genes for the summary statistics in Figure S5 and S11. Pvalues were calculated by using the union of the bootstrap values to calculate what proportion of the differences between the groups significantly differed from 0, multiplied by 2, to account for the fact that the test is one-sided.
