# Supplementary material for: Cis-regulatory evolution spotlights species differences in the adaptive potential of gene expression plasticity
Source: Nat Commun. 2021 Jun 7;12:3376. doi: 10.1038/s41467-021-23558-2 (PMC8184852; doi:10.1038/s41467-021-23558-2)
Supplement: Supplementary file 11 — Reporting Summary [file 41467_2021_23558_MOESM11_ESM.pdf]

Corresponding author(s): Juliette de Meaux
Last updated by author(s): 14/4/21

Reporting Summary

Nature Research wishes to improve the reproducibility of the work that we publish. This form provides structure for consistency and transparency in reporting. For further information on Nature Research policies, see our Editorial Policies and the Editorial Policy Checklist.

Statistics

For all statistical analyses, confirm that the following items are present in the figure legend, table legend, main text, or Methods section.

n/a	Confirmed	
☐	☒	The exact sample size (n) for each experimental group/condition, given as a discrete number and unit of measurement
☐	☒	A statement on whether measurements were taken from distinct samples or whether the same sample was measured repeatedly
☐	☒	The statistical test(s) used AND whether they are one- or two-sided<br>*Only common tests should be described solely by name; describe more complex techniques in the Methods section.*
☐	☒	A description of all covariates tested
☐	☒	A description of any assumptions or corrections, such as tests of normality and adjustment for multiple comparisons
☐	☒	A full description of the statistical parameters including central tendency (e.g. means) or other basic estimates (e.g. regression coefficient) AND variation (e.g. standard deviation) or associated estimates of uncertainty (e.g. confidence intervals)
☐	☒	For null hypothesis testing, the test statistic (e.g. F, t, r) with confidence intervals, effect sizes, degrees of freedom and P value noted<br>*Give P values as exact values whenever suitable.*
☐	☐	For Bayesian analysis, information on the choice of priors and Markov chain Monte Carlo settings
☐	☒	For hierarchical and complex designs, identification of the appropriate level for tests and full reporting of outcomes
☐	☒	Estimates of effect sizes (e.g. Cohen's d, Pearson's r), indicating how they were calculated

Our web collection on statistics for biologists contains articles on many of the points above.

Software and code

Policy information about availability of computer code

Data collection	No software was used to collect the data.
Data analysis	All softwares used for data analysis are described in the methods section of our manuscript.

For manuscripts utilizing custom algorithms or software that are central to the research but not yet described in published literature, software must be made available to editors and reviewers. We strongly encourage code deposition in a community repository (e.g. GitHub). See the Nature Research guidelines for submitting code & software for further information.

Data

Policy information about availability of data

All manuscripts must include a data availability statement. This statement should provide the following information, where applicable:
- Accession codes, unique identifiers, or web links for publicly available datasets
- A list of figures that have associated raw data
- A description of any restrictions on data availability

The sequencing data has been submitted to GENBANK under the study PRJNA640858. The Transcriptome Shotgun Assembly of Arabidopsis halleri has been deposited at DDBJ/EMBL/GenBank under the accession GJCZ00000000. The version described in this paper is the first version, GJCZ01000000.

Field-specific reporting

Ecological, evolutionary & environmental sciences study design

All studies must disclose on these points even when the disclosure is negative.

Study description	We report gene expression changes after 0, 1.5, 3, 6, 12 and 24h of dehydration stress in three species and 2 interspecific hybrid, replicated four times.
Research sample	Arabidopsis thaliana Col-0 genotype, A. lyrata MN47 genotype, A. halleri h2-2 genotype. All three chosen for their typical phenotypic response to stress and their genomic resources.
Sampling strategy	We performed the analyses on the basis of four replicates, to balance power to infer genetic differences and sequencing costs.
Data collection	We collected a transcriptome time series. The material for transcriptome sequencing was grown and collected by Fei He.
Timing and spatial scale	Four independent trials were performed in one week interval in the same growth chamber. Data was collected in 2015.
Data exclusions	No data was excluded from the analysis.
Reproducibility	We used four replicate to account for variance in estimates of gene expression. We verified that our data was reproducing previous studies of A. thaliana exposed to similar stress treatments, or of A. lyrata and A. halleri exposed to milder drought stress.
Randomization	Genotypes to be collected for each time point (6 plant for each genotypes) were fully randomized within each trial.
Blinding	Blinding was done at the time of RNA extraction. It is not possible at the time of sample collection because the plants look different.

Did the study involve field work? ☐ Yes ☒ No

Reporting for specific materials, systems and methods

We require information from authors about some types of materials, experimental systems and methods used in many studies. Here, indicate whether each material, system or method listed is relevant to your study. If you are not sure if a list item applies to your research, read the appropriate section before selecting a response.

Materials & experimental systems

n/a	Involved in the study
☒	☐ Antibodies
☒	☐ Eukaryotic cell lines
☒	☐ Palaeontology and archaeology
☒	☐ Animals and other organisms
☒	☐ Human research participants
☒	☐ Clinical data
☒	☐ Dual use research of concern

Methods

n/a	Involved in the study
☒	☐ ChIP-seq
☒	☐ Flow cytometry
☒	☐ MRI-based neuroimaging
